# *FOXO1* Gene Downregulation and Promoter Methylation Exhibits Significant Correlation With Clinical Parameters in Indian Breast Cancer Patients

**DOI:** 10.3389/fgene.2022.842943

**Published:** 2022-03-03

**Authors:** Mohammad Aasif Khan, Sheersh Massey, Irfan Ahmad, Sadaf, Naseem Akhter, Maria Habib, Saad Mustafa, S. V. S. Deo, Syed Akhtar Husain

**Affiliations:** ^1^ Human Genetics Laboratory, Department of Biosciences, Jamia Millia Islamia, New Delhi, India; ^2^ Department of Clinical Laboratory Sciences, College of Applied Medical Sciences, King Khalid University, Abha, Saudi Arabia; ^3^ Department of Laboratory Medicine, Faculty of Applied Medical Sciences, Albaha University, Albaha, Saudi Arabia; ^4^ Department of Surgical Oncology BRA-IRCH, All India Institute of Medical Sciences (AIIMS), New Delhi, India

**Keywords:** methylation, immunohistochemistry, clinical, tumor, diagnosis

## Abstract

**Background:** Forkhead box “O” one which is member of Forkhead box family of transcription factors is known to play key role in different physiological processes including cell cycle arrest, autophagy, and apoptosis. *FOXO1* is defined to play tumor suppressive role in various malignancies including breast cancer and its Dysregulation is frequently reported. However, the evaluation of *FOXO1* promoter methylation and its expression at mRNA and protein level in different stages of breast cancer and its association with different clinical parameters is still not studied. Therefore, for better understanding the role of *FOXO1* in breast cancer, in our study we examined the *FOXO1* mRNA and protein expression in Breast cancer samples of Indian breast cancer patients.

**Results:** Total 127 breast cancer samples along with adjacent normal tissue (*n = 127*) were analyzed through methylation specific PCR (MS-PCR), mRNA expression (Real-time PCR) and Immunohistochemistry (IHC). We detected 69.29% cases to be downregulated at the mRNA level, and 77.95% of cases exhibited no or low protein expression. In our data we report a significant association (*p = 0.0001*) between the downregulated protein expression and promoter hypermethylation of *FOXO1* gene. We also found a significant correlation of *FOXO1* mRNA level with Age (*p = 0.008*), age at first live birth (*p = 0,003*), tumor size (*p = 0.05*) and lymph node status (*p = 0.01*).

**Conclusion:** we in our study report the tumor suppressive role of *FOXO1* in case of Indian breast cancer patients and our data suggest it to exhibit prognostic importance. However, further research is needed to evaluate *FOXO1* significance in diagnostic and therapeutic targeting in breast cancer cases.

## Introduction

According to Global Cancer statistics 2020, there were 684,996 deaths reported due to breast malignancy and it showed the highest incidence among all cancers (Globocan 2020). Breast cancer is a multifaceted disease exhibiting diverse morphological and histopathological features (Viale, 2012). To recognize the potential genes and their molecular mechanism associated with the pathogenesis of the disease still needs to be investigated (Tang et al., 2018). Lack of early diagnosis and inadequate personalized approach in treatment are chief factors in terms of poor survival of the patients (Shi et al., 2018). Hence, there is a necessity in the current time to search for more reliable molecular targets to develop a better diagnostic and therapeutic approach in the treatment of breast cancer (Chan et al., 2017; Jiang et al., 2018). Therefore, our study focuses on molecular profiling of the Forkhead box O 1 (*FOXO1*) gene which is a potent molecule and can exhibit promising results in the development of diagnostic and therapeutic strategies.

*FOXO1* is one of the key members of the *FOXO* transcription factors subfamily, which is located on chromosome 13 (13q14.11) and is a chief target of insulin signalling. It is known to have a major role in the regulation of metabolic homeostasis, autophagy, apoptosis, cell cycle arrest genes, and immune regulators (Xing et al., 2018; Jiang et al., 2018; Kousteni, 2012, Gene cards). The activation of *FOXO1* via binding of insulin or several growth factors to their receptors consequently activates PI3K (phosphoinositide kinase) that further triggers the activity of other kinases including Akt and SGK (serum glucocorticoid inducible kinase). However, in the absence of insulin, the *FOXO1* is found to have nuclear localization and leads to cell cycle arrest. Thus, in presence of insulin or IGF-1, PI3K/Akt/SGK pathway is directly activated while *FOXO1* is inhibited resulting in cell survival (Cantley, 2002; Li et al., 2015). The low levels of *FOXO1* have been linked with tumor progression in several recent studies (Kaymaz et al., 2017; Procaccia et al., 2017). The decreased nuclear and cytoplasmic expressions of *FOXO1* in the case of breast cancer have been reported in the previous studies (Wu et al., 2012). However, the correlation of lower levels of *FOXO1* at mRNA and protein level with clinical parameters is not well known. The current study proposes to determine the correlation between methylation and expression of FOXO1 in breast cancer biopsy as compared to adjacent normal tissue.

## Methodology and Materials

### Collection of Biological Specimens

In our study, 127 participants were enrolled, and the cancerous tissue along with the adjacent non-cancerous tissue of the breast was obtained and stored at −20° for further experiments and analysis. The inclusion criteria for the specimens in the study included the histopathologically confirmed breast cancer patients of age group 20–79 years having at least 6 months of life expectancy.

Following clinical parameters were included for the study such as tumor size, age at diagnosis, histological grade, the status of lymph node (LN), reproductive history, and information (age at menarche and menopausal status), clinical staging or TNM stages, Estrogen receptor (ER) (+ or -), Progesterone receptor (PR) (+ or -) and Human Epidermal Growth Factor Receptor 2 (HER2) (+ or -).

The females (*n = 127*) included in the study were clinically confirmed with sporadic breast cancer and were genetically unrelated. Normal adjacent breast tissue was taken as control.

### Inclusion Criteria

The study involved females with histopathologically confirmed primary breast cancer and having at least 6 months of life expectancy, lying between the age group 20–79 years. The participants provided consent to abide by the procedures of the study. All the females included in the study were registered in the medical record book of AIIMS, New Delhi, and their medical records were evaluated for studying various clinical and pathological parameters of the patients.

### Real-Time Polymerase Chain Reaction

For the isolation of RNA, the breast cancer tissues, and the collected normal tissues were preserved in the RNAlater (Qiagen) kit, and afterward, RNA isolation was done by using TRIzo1Reagent (Invitrogen) by following the instruction provided by the manufacturer in the protocol. Thereafter, the complementary DNA (cDNA) from total RNA was synthesized using a cDNA kit (verso Thermo Fisher Scientific) and later storage was done at −20°C for further analysis. Subsequently, the above-prepared cDNA was used in the (qPCR) where amplification was carried out using Roche Light Cycler^®^ 96 SYBR Green I Master mix. By applying the primers for *FOXO1*: sense 5′- CCA​CAT​TCA​ACA​GGC​AGC​AG-3′ antisense 5′- GAC​GGA​AAC​TGG​GAG​GAA​GG-3′ which amplified a 152-bp. product. *β* actin gene was taken as an internal control and amplified in the same qPCR reaction. The primers used for qPCR reaction were sense 5′-AGA​TAG​TGG​ATC​AGC​AAG​CAG-3′ and antisense 5′-GCG​AAG​TTA​GGT​TTT​GTC​A-3′, which amplified a 160 bp. product. Standardized protocol of our laboratory (Real et al., 2018; Sadaf et al., 2018; Khan et al., 2020) was used to perform PCR. Measurements were taken in triplicates. The calculation was done for the relative amount of mRNA using Light Cycler 96 (Roche) equipped with Software 1.5. The calibrated normalized ratio was estimated as per the given standard formula: RQ = 2-∆∆Cq = [(Cq targeted gene–Cq βactin) calibration sample].

### DNA Extraction

Phenol-chloroform Isoamyl (PCI) method was used for isolation of gDNA from Breast cancer and adjacent normal tissue (Russell and Sambrook, 2001). The quantity and quality evaluation of isolated genomic DNA was done using a Nanodrop spectrophotometer (ND1000), and agarose gel electrophoresis was further performed for validation.

### Methylation Through MS-PCR

**FIGURE 1:**
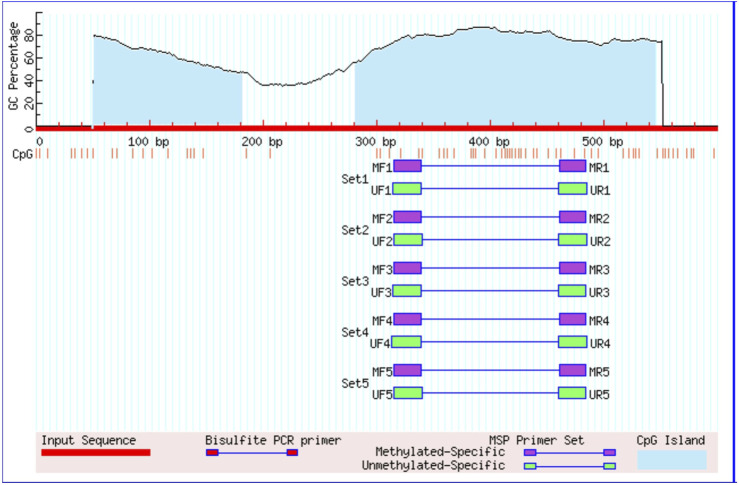
Graphical represntation of CpG islands in the foxo1 promoter region taken from Meth Primer. Criteria used: Island size>100, GC Percent >50.0, Obs/Exp>0.60/.

EZ DNA Methylation-Gold™ Kit was used to carry out Bisulfite conversion following the instruction given by the manufacturer. The converted product was amplified using dual sets of methylated and unmethylated *FOXO1* primers. Eukaryotic promoter database was used to retrieve the *FOXO1* gene promoter sequence, and MethPrimer software was used for primer designing (Figure 1). When searched by MethPrimer, the promoter region of the *FOXO1* gene was found to contain two CpG islands of 600 bp. The primer pairs that were used for the detecting methylation in the promoter region of *FOXO1* were as follows: sense 5′- GGA​AAA​TCG​GGT​TTT​ATT​TAG​TTC-3′ and antisense 5′- GAC​TAC​TAC​GAC​TAC​CAA​ACC​GC-3′, for the unmethylated detection: sense 5′- TGG​AAA​ATT​GGG​TTT​TAT​TTA​GTT​T-3 and antisense 5′- CAA​CTA​CTA​CAA​CTA​CCA​AAC​CAC​C-3′. The product size for unmethylation was 172 bp, and for methylation, it was 170 bp. MS-PCR was done by the following condition: initially denaturation at 95°C for 5 min, 35 cycles of amplification at 95°C for the 30 s, annealing at 53.9°C (methylation) and 52.7°C (unmethylation) for 30 s, 72°C for 30 s and final extension at 72°C for 7 min. The pictures of the amplified product were obtained on a 2% agarose gel having EtBr, visualization was done under ultraviolet (UV) illumination with the Gel Doc (Bio-Rad Molecular Imaging System). The experiments were executed in triplicate without any disparity observed among the replicates.

### Immunohistochemistry

Breast cancer tissue and adjacent normal tissue stored in formalin were used for Block preparation. Poly-l-lysine slides were used, and the sections of the block were taken on slides. Subsequently, different grades of xylene were used for deparaffinization, and rehydration was done using ethanol. The quenching of internal peroxide activity was done by 0.3% hydrogen peroxide, and citrate buffer boiling resulted in Ag withdrawal. To non-specific interaction of protein was ceased using serum solution as a blocking agent, and incubation at 4°C with primary antibody (CST#2880 *FOXO1*, 1:100) was done overnight. Furthermore, anti-rabbit biotinylated secondary antibody and streptavidin HRP incubation was done for 20–30 min, respectively. 3,3́- 3,3′-Diaminobenzidine (DAB) method was used to visualize antibody binding sites. Also, Hematoxylin counterstaining was carried out. For positive control, normal breast tissue was considered, while for the negative control, the primary antibody was skipped following the same protocol resulting in no staining. Interpretation of the staining was carried out under the guidance expert histopathologists using light microscope (magnification ×400), and the grading was done as follows: [1] 0% tumor staining with no expression, [2] 1%–10% tumor staining with mild expression (+), [3] 10%–50% tumor staining denoting moderate expression (++) [4] >50% tumor staining indicating high expression (+++ or ++++).

### Statistical Analysis

SPSS-IBM (version 22.0) was utilized to find the relevant association with the clinicopathological parameters. The current study data are represented as mean ± standard error (SE). the *p*-value of less than 0.005 was considered significant. To evaluate the significance of differential *FOXO1* mRNA expression levels, a non-parametric test, i.e., Wilcoxon signed-rank test was used.

## Results

### Downregulated *FOXO1* mRNA Expression in Breast Cancer Cases and its Correlation With Clinicopathological Parameters

**FIGURE 2:**
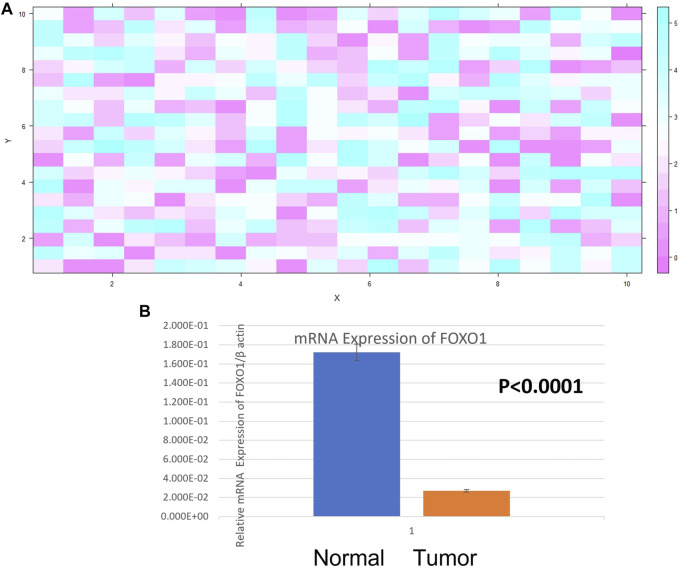
**(A)** Heat Map plot (analyzed by R platform version 3.6.3 64-bit) of FOXO1 mRNA relative expression (fold change) in Breast cancer cases. *X*-axis depicts Δ Cq target against *Y*-axis Δ Cq control at default parameters. **(B)** Relative mRNA expression of FOXO1/*β* ACTIN in Breast tumor and adjacent normal tissue.

The expression of *FOXO1* at the mRNA level was detected in case of breast cancer and adjacent normal tissue. Its expression was normalized against beta-actin expression. *FOXO1* mRNA expression was found to be downregulated in 69.29% cases (88/127) and out of which 73.80% cases (65/88) were categorized under histological grade I and II of breast cancer. The fold change of 88 downregulated cases was examined to be 5.16 the expression of *FOXO1* in breast cancer tissue was 1.16 ± 0.02 (Mean ± SE) and in the normal tissue was 1.95 ± 0.07 (Mean ± SE) (*p < 0.0001*). Correlating the *FOXO1* mRNA expression with different clinic pathological parameters of patients indicated significant association with Age, Age at first live birth, Tumor Size, Lymph Node Status (Table 1, Table 2, Figure 2).

**TABLE 1:** Characteristics of study subjects (*n =127*).

S.no	Characteristic	Cases (%)
1	Age (years)	
	≤50	44 (34.65)
	>50	83 (65.35)
2	Geographic location	
	Rural	33 (25.98)
	Urban	94 (74.02)
3	Age at menarche	
	≤12	20 (15.75)
	>12	107 (84.25)
4	Age at first live birth	
	≤25	100 (78.75)
	>25	27 (21.25)
5	Breast feeding	
	Yes	122 (96.06)
	No	5 (3.94)
6	Use of exogenous hormone	
	Yes	6 (4.72)
	No	121 (95.28)
7	Family history of cancer	
	Yes	21 (16.54)
	No	106 (83.46)
8	Menopausal status	
	Premenopausal	36 (28.35)
	Postmenopausal	91 (71.65)
9	Age at menopause	
	≤45	39 (42.86)
	>45	52 (57.14)
10	ER status	
	Positive	92 (72.44)
	Negative	35 (27.56)
11	PR status	
	Positive	64 (50.39)
	Negative	63 (49.61)
12	Her2 status	
	Positive	61 (48.03)
	Negative	66 (51.97)
13	Molecular subtypes (Breast cancer)	
	Luminal A	45 (35.43)
	Luminal B	51 (40.16)
	Her2 enriched	17 (13.38)
	Triple negative breast cancer (TNBC)	14 (11.03)
14	Tumor size	
	≤5	59 (46.46)
	>5	68 (53.54)
15	Lymph node status	
	Positive	109 (85.83)
	Negative	18 (14.17)
16	TNM stage	
	I + II	36 (28.35)
	III + IV	91 (71.65)
17	Histological grade	
	I + II	102 (80.31)
	III	25 (19.69)

**TABLE 2:** Correlation study of *FOXO1* mRNA expression levels with clinical parameters of Breast Cancer case.

Characteristics	Total (*n= 127*)	*FOXO1* mRNA expression relative to beta actin (Mean ± S.E)	*p-Value*	Chi-squared
Age				
<50	44 (34.65)	0.58 ± 0.01		
≥50	83 (65.35)	0.94 ± 0.02	0.008*	6.93
Geographical location				
Rural	33 (25.98)	1.66 ± 0.02		
Urban	94 (74.02)	1.70 ± 0.21	0.95	0.003
Age of menarche				
≤12	20 (15.75)	1.21 ± 0.04		
>12	107 (84.25)	1.67 ± 0.01	0.13	2.27
Age at first live birth				
≤25	100 (78.74)	1.32 ± 0.05		
>25	27 (21.26)	1.20 ± 0.06	0.0003*	13.13
Breast feeding				
Yes	122 (96.06)	1.30 ± 0.02		
No	5 (3.94)	0.71 ± 0.01	0.64	0.21
Use of exogenous hormone				
Yes	6 (4.72)	1.37 ± 0.07		
No	121 (95.28)	1.19 ± 0.08	0.88	0.20
Family history of cancer				
Yes	21 (16.54)	0.91 ± 0.07		
No	106 (83.46)	1.70 ± 0.09	0.42	0.64
Menopausal Status				
Premenopausal	36 (28.34)	0.92 ± 0.00		
Postmenopausal	91 (71.66)	1.23 ± 0.01	0.40	0.68
Age at Menopausal				
≤45	39 (42.85)	1.78 ± 0.08		
>45	52 (57.15)	1.23 ± 0.02	0.94	0.004
Estrogen receptor status				
Negative	35 (27.56)	0.83 ± 0.02		
Positive	92 (72.44)	1.38 ± 0.21	0.58	0.29
Progesterone receptor status				
Negative	63 (49.61)	1.47 ± 0.33		
Positive	64 (50.39)	1.11 ± 0.04	0.36	0.81
Her2 neu Status				
Negative	66 (51.97)	1.18 ± 0.03		
Positive	61 (48.03)	1.74 ± 0.12	0.77	0.07
Tumor Size				
<5	68 (53.54)	1.17 ± 0.07		
≥5	59 (46.46)	1.21 ± 0.09	0.05*	3.54
Lymph Node Status				6.05
Positive	109 (85.83)	1.50 ± 0.01	0.01*	
Negative	18 (14.17)	0.88 ± 0.06		
TNM Staging				
Stage (I + II)	36 (28.35)	1.17 ± 0.001		
Stage (III + IV)	91 (71.65)	1.98 ± 0.02	0.20	1.58
Histological Grade				
(I + II)	102 (80.31)	2.01 ± 0.01	0.74	0.10
(III)	25 (19.69)	1.47 ± 0.07		
Molecular Subtypes				
Luminal A	45 (35.43)	1.52 ± 0.12		
Luminal B	51 (40.16)	1.33 ± 0.30	0.89	0.59
Her2neu Enriched	17 (13.38)	0.47 ± 0.01		
TNBC	14 (11.03)	1.21 ± 0.11		

TNBC, triple negative breast cancer; *FOXO1*, Forkhead Box O1 ^a^ Only Downregulated Cases were included.

### *FOXO1* Promoter Methylation and its Correlation With Clinical Parameters of Patients

**FIGURE 3:**
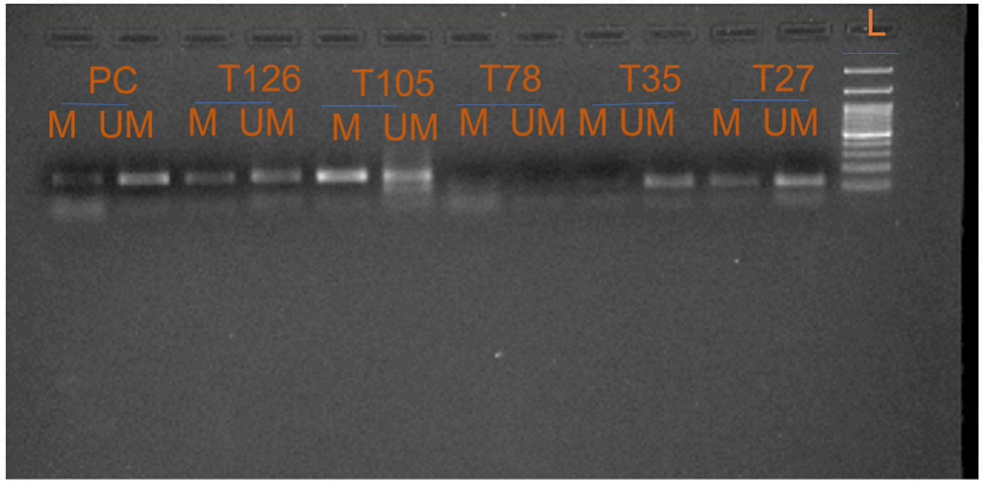
Representative gel picture of Methylation—specific PCR analysis of FOXO1 gene in Breast cancer patients: DNA methylation was assessed using two specifically designed primers to amplify either methylated DNA (M) or unmethylated DNA (UM) (L: 100 bp DNA ladder; number indicates the case number; PC: Positive Control; T: Tumour tissue).

Promoter methylation study of *FOXO1* promoter region was done through Methylation Specific PCR, the hypermethylated promoter region of *FOXO1* was found in 61.41% (78/127) cases. The correlation of promoter methylation with clinical parameters revealed a significant association with the menopausal status of breast cancer patients. In progressive stages III and IV of breast cancer, 58/91 cases were found to be methylated (Figure 3; Table 3.)

**TABLE 3:** Correlation study of *FOXO1* Promoter Methylation status with clinical parameters of Breast Cancer Patients.

Characteristics	Total cases (*n = 127*)	Methylated	Unmethylated	*p-Value*	Chi-squared
Age					
<50	44 (34.65)	29 (65.90)	15 (34.10)	0.44	0.57
≥50	83 (65.35)	49 (59.04)	34 (40.96)		
Geographical location					
Rural	33 (25.98)	19 (57.57)	14 (42.43)	0.59	0.27
Urban	94 (74.02)	59 (62.76)	35 (37.24)		
Age of menarche					
≤12	20 (15.75)	13 (65.00)	07 (35.00)	0.71	0.12
>12	107 (84.25)	65 (60.74)	42 (39.26)		
Age at first live birth					
≤25	100 (78.74)	57 (100)	43 (43)	0.04	3.87
>25	27 (21.26)	21 (77.77)	6 (22.22)		
Breast feeding					
Yes	122 (96.06)	75 (61.47)	47 (38.53)	0.94	0.004
No	5 (3.94)	03 (60)	2 (40)		
Use of exogenous hormone					
Yes	6 (4.72)	04 (66.66)	02 (33.33)	0.78	0.07
No	121 (95.28)	74 (61.15)	47 (38.85)		
Family history of cancer					
Yes	21 (16.54)	14 (66.66)	07 (33.33)	0.58	0.29
No	106 (83.46)	64 (60.37)	42 (39.63)		
Menopausal Status					
Premenopausal	36 (28.34)	27 (75)	09 (25)	0.04*	3.91
Postmenopausal	91 (71.66)	51 (56.04)	40 (43.96)		
Age at Menopausal					
≤45	39 (42.85)	23 (58.97)	16 (41.03)	0.66	0.19
>45	52 (57.15)	33 (63.46)	19 (36.54)		
Estrogen receptor status					
Negative	35 (27.56)	25 (71.42)	10 (28.58)	0.15	2.04
Positive	92 (72.44)	53 (57.60)	39 (42.40)		
Progesterone receptor status					
Negative	63 (49.61)	40 (63.49)	23 (36.51)	0.63	0.22
Positive	64 (50.39)	38 (59.37)	26 (40.63)		
Her2 neu Status					
Negative	66 (51.97)	43 (65.15)	23 (34.85)	0.36	0.80
Positive	61 (48.03)	35 (57.38)	26 (42.62)		
Tumor Size					
<5	68 (53.54)	46 (67.65)	22 (32.35)	0.12	2.39
≥5	59 (46.46)	32 (54.23)	27 (45.7)		
Lymph Node Status					
Positive	109 (85.83)	65 (59.63)	44 (40.37)	0.30	1.03
Negative	18 (14.17)	13 (72.23)	05 (27.77)		
TNM Staging					
Stage (I + II)	36 (28.35)	20 (55.55)	16 (44.46)	0.39	0.72
Stage (III + IV)	91 (71.65)	58 (63.74)	33 (36.26)		
Histological Grade					
(I + II)	102 (80.31)	60 (58.82)	42 (41.18)	0.22	1.47
(III)	25 (19.69)	18 (72)	07 (28)		
Molecular Subtypes					
Luminal A	45 (35.43)	26 (57.78)	19 (42.22)	0.66	1.59
Luminal B	51 (40.16)	30 (58.83)	21 (41.17)		
Her2neu Enriched	17 (13.38)	12 (70.59)	05 (29.41)		
TNBC	14 (11.03)	10 (71.43)	04 (28.57)		

### Low or No Expression of *FOXO1* Protein in Breast Cancer Tissue

**FIGURE 4:**
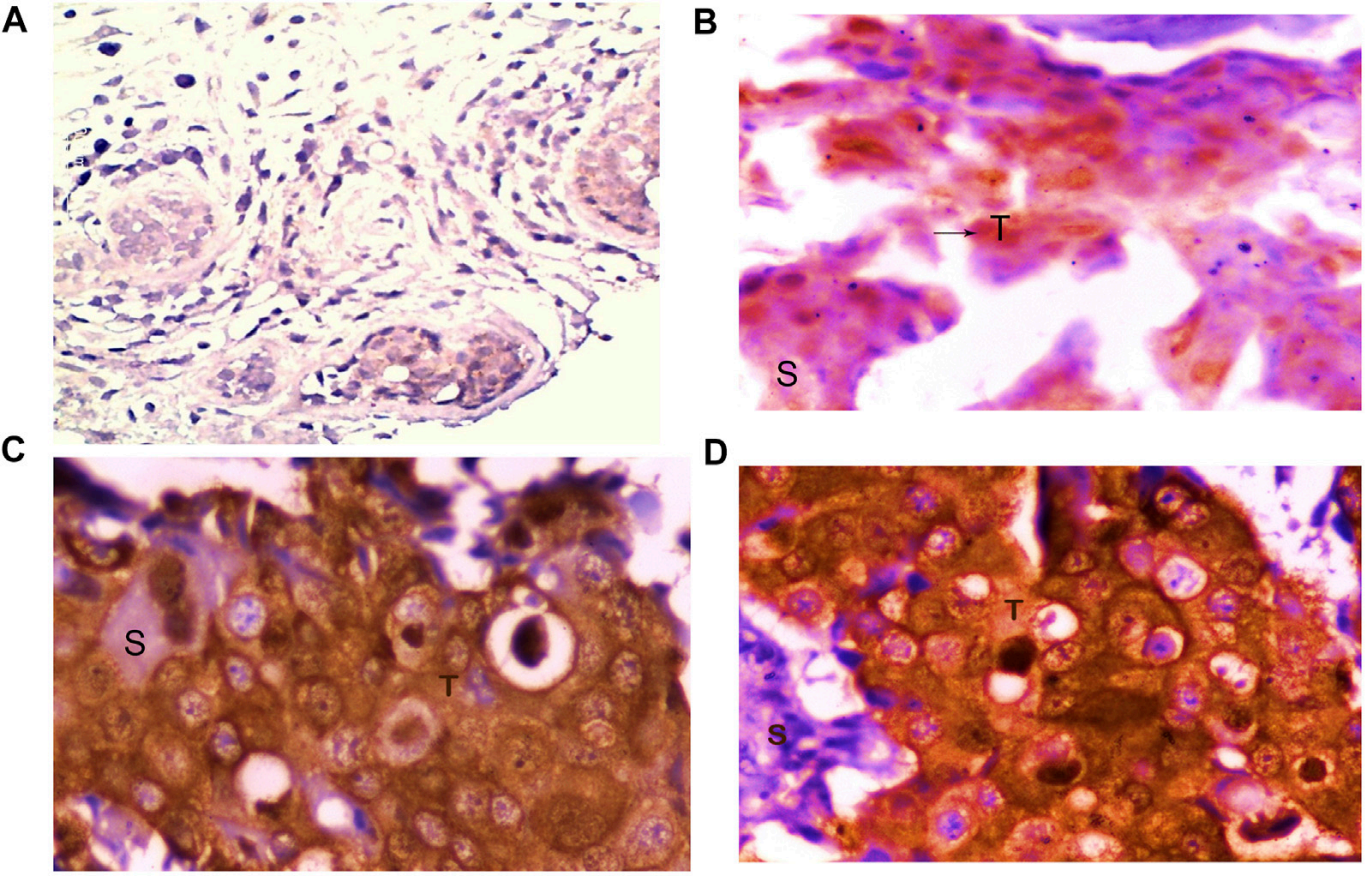
Representative picture of Immunohistochemical staining of human breast cancer tissue samples by anti-FOXO1 antibody (magnification: ×400) showing **(A)** no expression, **(B)** low (+) expression, **(C)** moderate (++) expression, and **(D)** high (+++) expression of FOXO1. S: stromal tissue, T: tumor tissue.

*FOXO1* expression analysis at the protein level was done by IHC and it was found to be absent in 77.95% (99/127) cases. However, 28 Cases exhibited the moderate or high expression of *FOXO1* protein. Also, the *FOXO1* protein expression pattern substantiated the mRNA expression. Furthermore, the percentage of *FOXO1* protein downregulation was significant with Her2 neu status, tumor size, and histological grade of breast cancer (Figure 4; Table 4.)

**TABLE 4:** Correlation of *FOXO1* Protein Expression level with clinical parameters of Breast Cancer Patients.

Characteristics	Total cases (*n = 127*)	*FOXO1* absent	*FOXO1* present	*p Value*	Chi-squared
Age					
<50	44 (34.65)	36 (81.81)	08 (18.19)	0.44	0.58
≥50	83 (65.35)	63 (75.90)	20 (24.10)		
Geographical location					
Rural	33 (25.98)	29 (87.87)	04 (12.13)	0.10	2.56
Urban	94 (74.02)	70 (74.46)	24 (25.54)		
Age of menarche					
≤12	20 (15.75)	16 (80)	04 (20)	0.80	0.05
>12	107 (84.25)	83 (77.57)	24 (22.43)		
Age at first live birth					
≤25	100 (78.74)	78 (78)	22 (22)	0.98	0.001
>25	27 (21.26)	21 (77.77)	06 (22.23)		
Breast feeding					
Yes	122 (96.06)	96 (78.68)	26 (21.32)	0.32	0.97
No	5 (3.94)	03 (60)	02 (40)		
Use of exogenous hormone					
Yes	6 (4.72)	02 (33.33)	04 (66.66)	0.006*	7.29
No	121 (95.28)	97 (80.16)	24 (19.84)		
Family history of cancer					
Yes	21 (16.54)	18 (85.72)	03 (14.28)	0.34	0.88
No	106 (83.46)	81 (76.42)	25 (23.58)		
Menopausal Status					
Premenopausal	36 (28.34)	32 (88.88)	04 (11.12)	0.06	3.49
Postmenopausal	91 (71.66)	67 (73.62)	24 (26.38)		
Age at Menopausal					
≤45	39 (42.85)	25 (64.10)	14 (35.90)	0.80	0.06
>45	52 (57.15)	32 (61.53)	20 (38.47)		
Estrogen receptor status					
Negative	35 (27.56)	28 (80)	07 (20)	0.73	011
Positive	92 (72.44)	71 (77.17)	21 (22.83)		
Progesterone receptor status					
Negative	63 (49.61)	50 (79.36)	13 (20.64)	0.70	0.14
Positive	64 (50.39)	49 (76.56)	15 (23.44)		
Her2 neu Status					
Negative	66 (51.97)	58 (87.87)	08 (13.79)	0.005*	7.87
Positive	61 (48.03)	41 (67.21)	20 (32.79)		
Tumor Size					
<5	68 (53.54)	59 (86.76)	09 (13.24)	0.01*	6.61
≥5	59 (46.46)	40 (67.80)	19 (32.20)		
Lymph Node Status					
Positive	109 (85.83)	84 (77.06)	25 (22.94)	0.55	0.35
Negative	18 (14.17)	15 (83.33)	03 (16.67)		
TNM Staging					
Stage (I + II)	36 (28.35)	25 (69.44)	11 (30.56)	014	2.11
Stage (III + IV)	91 (71.65)	74 (81.31)	17 (18.69)		
Histological Grade					
(I + II)	102 (80.31)	76 (74.50)	26 (25.50)	0.05*	3.72
(III)	25 (19.69)	23 (92.00)	02 (8.00)		
Molecular Subtypes					
Luminal A	45 (35.43)	35 (77.77)	10 (22.23)	0.50	2.31
Luminal B	51 (40.16)	42 (82.35)	09 (17.65)		
Her2neu Enriched	17 (13.38)	11 (64.70)	06 (35.30)		
TNBC	14 (11.03)	11 (78.57)	03 (21.43)		

### *FOXO1* Promoter Methylation and its Association With Protein Expression

The results represented a strong correlation of *FOXO1* protein expression with the promoter methylation and 74 out of 78 hypermethylated cases showed low or no protein expression and 04 cases showed protein expression. In 51.02% (25/49) cases that showed no methylation had no protein expression. The cases which had downregulation of *FOXO1* showed 78.40% (69/88) hypermethylation while 23.07% (9/39) cases had moderate to high-level protein expression. highly significant *p*-value (*p= 0.0001*) was found between *FOXO1* methylation in the promoter region and protein expression, which represented a strong correlation Tables 5,6,7

**TABLE 5:** Correlation study of Promoter Methylation with Protein expression in Breast Cancer Patients from North India.

*FOXO1* promoter	*FOXO1* protein expression		Total (%)	*p* Value	Chi-squared
	Absent	Present			
Methylated	74 (94.87)	04 (5.13)	78 (61.41)		
Unmethylated	25 (51.02)	24 (48.98)	49 (38.59)	0.0001	33.67
Total	99 (77.95)	28 (22.05)	127		

*p* Value (Fischer’s Exact Test).

**TABLE 6:** Correlation study of methylation and protein expression in samples having methylated *FOXO1* promoter or *FOXO1* expression loss with clinical parameters of Breast cancer patients from North Indian population.

Clinical characteristics		Total methylated (*n* = 78)	Methylated *FOXO1*		*p* Value	Chi-squared	Total (N)	*FOXO1* loss		*p* Value	Chi-squared
			*FOXO1* absent	*FOXO1* present				Methylated *FOXO1*	Unmethylated *FOXO1*		
Age											
<50	44 (34.65)	29	26	03	0.10	2.58	36	26	10	0.66	0.91
≥50	83 (65.35)	49	48	01			63	48	15		
Geographical location											
Rural	33 (25.98)	19	17	02	0.22	1.50	29	17	12	0.01*	5.65
Urban	94 (74.02)	59	57	02			70	57	13		
Age of menarche											
≤12	20 (15.75)	13	10	03	0.001*	10.33	16	10	06	0.21	1.51
>12	107 (84.25)	65	64	01			83	64	19		
Age at first live birth											
≤25	100 (78.74)	57	56	01	0.02*	4.95	78	56	22	0.19	1.69
>25	27 (21.26)	21	18	03			21	18	03		
Breast feeding											
Yes	122 (96.06)	75	72	03	0.02*	5.10	96	72	24	0.74	0.10
No	5 (3.94)	03	02	01			03	02	01		
Use of exogenous hormone											
Yes	6 (4.72)	04	02	02	0.0001*	17.44	02	02	00	0.40	0.69
No	121 (95.28)	74	72	02			97	72	25		
Family history of cancer											
Yes	21 (16.54)	14	11	03	0.002	9.31	18	11	07	0.14	2.16
No	106 (83.46)	64	63	01			81	63	18		
Menopausal Status											
Premenopausal	36 (28.34)	27	26	01	0.67	0.17	32	26	06	0.30	1.05
Postmenopausal	91 (71.66)	51	48	03			67	48	19		
Age at Menopausal											
≤45	39 (42.85)	23	21	02	0.95	0.003	25	21	04	0.23	1.41
>45	52 (57.15)	33	30	03			32	30	02		
Estrogen receptor status											
Negative	35 (27.56)	25	24	01	0.75	0.09	28	24	04	0.11	2.48
Positive	92 (72.44)	53	50	03			71	50	21		
Progesterone receptor status											
Negative	63 (49.61)	40	39	01	0.28	1.16	50	39	11	0.45	0.56
Positive	64 (50.39)	38	35	03			49	35	14		
Her2 neu Status											
Negative	66 (51.97)	43	42	01	0.21	1.54	58	42	16	0.52	0.40
Positive	61 (48.03)	35	32	03			41	32	09		
Tumor Size											
<5	68 (53.54)	46	44	02	0.70	0.14	59	44	15	0.96	0.002
≥5	59 (46.46)	32	30	02			40	30	10		
Lymph Node Status											
Positive	109 (85.83)	65	62	03	0.64	0.21	84	62	22	0.61	0.25
Negative	18 (14.17)	13	12	01			15	12	03		
TNM Staging											
Stage (I + II)	36 (28.35)	20	19	01	0.97	0.001	25	19	06	0.86	0.02
Stage (III + IV)	91 (71.65)	58	55	03			74	55	19		
Histological Grade											
(I + II)	102 (80.31)	60	58	02	0.18	1.72	76	58	18	0.51	0.42
(III)	25 (19.69)	18	16	02			23	16	07		
Molecular Subtypes											
Luminal A	45 (35.43)	26	25	01	0.79	1.02	35	25	10	0.07	6.89
Luminal B	51 (40.16)	30	28	02			42	28	14		
Her2neu Enriched	17 (13.38)	12	11	01			11	11	00		
TNBC	14 (11.03)	10	10	00			11	10	01		

**TABLE 7:** Correlation analysis between *FOXO1* methylation and *FOXO1* protein expression in stratification by various clinical characteristics of Breast cancer patients from North India.

Clinical characteristics	Total methylated (*n = 78*)	*FOXO1* methylation status	*FOXO1* expression		*p* Value	Chi-squared
			Absent	Present		
Age						
<50 44(34.65)	29	M	26	03
		U	10	05	0.06	3.51
≥50 83(65.35)	49	M	48	01		
		U	15	19	0.0001*	31.84
Geographical location						
Rural 33(25.98)	19	M	17	02		
		U	12	02	0.74	0.10
Urban 94(74.02)	59	M	57	02		
		U	13	22	0.12	2.31
Age of menarche						
>12,107(84.25)	65	M	64	01		
		U	19	23	0.0001*	41.54
≤12 20(15.75)	13	M	10	03		
		U	06	01	0.63	0.22
Age at first live birth						
≤25,100(78.74)	57	M	56	01		
		U	22	21	0.0001*	31.66
>25 27(21.26)	21	M	18	03		
		U	03	01	0.59	0.28
Breast feeding						
Yes 122(96.06)	75	M	72	03		35.26
		U	24	23	0.0001*	
No 5(3.94)	03	M	02	01		0.13
		U	01	01	0.70	
Use of exogenous hormone						
Yes 6(4.72)	04	M	02	02		1.50
		U	00	02	0.22	
No 121(95.28)	74	M	72	02		
		U	25	22	0.0001*	35.16
Family history of cancer						
Yes 21(16.54)	14	M	11	03	0.18	1.75
		U	07	00		
No 106(83.46)	64	M	63	01	0.0001*	43.46
		U	18	24		
Menopausal Status						
Premenopausal 36(28.34)	27	M	19	01		
		U	06	10	0.0002*	13.85
Postmenopausal 91(71.66)	51	M	55	03		
		U	18	15	0.0001*	21.50
Age at Menopausal						
≤45 39(42.85)	23	M	21	02		18.02
		U	04	12	0.0001*	
>45 52(57.15)	33	M	30	03		
		U	02	17	0.0001*	32.91
Estrogen receptor status						
Negative 35(27.56)	25	M	24	01	0.0002*	14.00
		U	04	06		
Positive 92(72.44)	53	M	50	03		
		U	21	18	0.0001*	20.91
Progesterone receptor status						
Negative 63(49.61)	40	M	39	01	0.0001*	22.00
		U	11	12		
Positive 64 (50.39)	38	M	35	03		
		U	14	12	0.0004*	12.59
Her2 neu Status						
Negative 66(51.97)	43	M	42	01		
		U	16	07	0.0009*	11.11
Positive 61(48.03)	35	M	32	03		
		U	09	17	0.0001*	21.85
Tumor Size						
<5 68(53.54)	46	M	44	02		
		U	15	07	0.001*	9.78
≥5 59(46.46)	32	M	30	02		
		U	10	17	0.0001*	21.57
Lymph Node Status						
Positive 109(85.83)	65	M	63	03		
		U	22	21	0.0001*	29.74
Negative 18(14.17)	13	M	12	01		
		U	03	02	0.09	2.71
TNM Staging						
Stage (I + II) 36(28.35)	20	M	19	01		
		U	06	10	0.0002*	13.85
Stage (III + IV) 91 (71.65)	58	M	55	03		
		U	19	14	0.0001*	19.21
Histological Grade						
(I + II) 102(80.31)	60	M	58	02		
		U	18	24	0.0001*	37.66
(III) 25(19.69)	18	M	16	02		
		U	07	00	0.35	0.84
Molecular Subtypes						
		M	25	01	0.0005*	12.03
Luminal A 45(35.43)	26	U	10	09		
		M	28	02		
Luminal B 51(40.16)	30	U	14	07	0.01*	6.04
		M	11	01		
Her2neu Enriched 17(13.38)	12	U	00	05	0.0003*	12.98
		M	10	00		
TNBC 14(11.03)	10	U	01	03	0.002*	9.54

## Discussion

*FOXO* subfamily of forkhead box transcription factor comprises four *FOXO* isoforms. *FOXO1* which is a member of this subfamily is the key target of insulin that inhibits its transcriptional events through nuclear exclusion (Kousteni, 2012). The prominent role of *FOXO1* is studied in the maintenance of tissue homeostasis at the time of various physiological as well as pathological conditions (Xing et al., 2018). In previous studies, the close association of lower *FOXO1* levels with human cancers such as hepatocellular carcinoma, colorectal cancer, pancreatic cancer, prostate cancer, and lung cancer have been demonstrated (Wu et al., 2012; Schwartz and Cote, 2016; Lou et al., 2017; Moeinifard et al., 2017).

In the current study, we examined the expression level of *FOXO1* in 127 breast cancer tissues taken along with adjacent normal tissues from the Indian female breast cancer patients. To analyze the *FOXO1* mRNA expression, we performed real-time PCR. Further, we studied *FOXO1* protein expression and its subcellular localization through immunohistochemistry and the epigenetic modulation in the promoter region of the *FOXO1* was analyzed using MS-PCR. While investigating our data for *FOXO1* mRNA expression we found 88 out of 127 cases (69.29%) exhibiting the downregulation at the *FOXO1* mRNA level. The downregulation of *FOXO1* mRNA in our data links positively to the previous studies which state the tumor suppressive role of the *FOXO1* gene in cancer progression (Myatt et al., 2010; Prasad et al., 2014). Further, on correlating our results we found a strong correlation of *FOXO1* mRNA expression with the age of the patient (*p = 0.008*), age at first live birth (*p = 0.0003*), tumor size (*p = 0.05*), and lymph node status (*p = 0.01*) of the breast cancer patients. *FOXO1* mRNA downregulation was earlier reported to show a significant association with the lymph node status and age of the patients in prostate cancer cases (Yang et al., 2021), our study also reveals this strong correlation of *FOXO1* mRNA expression with these clinical parameters in case of breast cancer patients.

The results of our study to detect the protein expression and localization of *FOXO1* protein reveals 77.95% (99/127) cases having low or no expression of *FOXO1* protein, whereas 22.05% (28/127) show moderate or high expression. The anti-proliferative role of *FOXO1* has been reported in case of cervical and prostate cancer, moreover, the enforced expression of *FOXO1* in endometrioid endometrial cancer cells and SiHa cells blocked the cell proliferation and decreased the tumorigenic activity (Goto et al., 2008; Zhang et al., 2015; Yang et al., 2021). Our results also suggest the tumor suppressive role of *FOXO1* in breast cancer and show a strong association of low protein expression with the histological grade (*p = 0.05*) and tumor size (*p = 0.01*) of breast cancer patients. The downregulated expression of *FOXO1* protein in the advanced stages (III and IV) of breast cancer suggests its repressive role in tumor progression and can be considered as the prognostic marker. Further, our investigation of *FOXO1* gene promoter methylation represented the hypermethylation (61.41%) of the *FOXO1* promoter region in most cases and a significant association (*p = 0.0001*) was found between the promoter methylation and protein expression of the *FOXO1* gene. (Table 5, 6). Also, the *FOXO1* promoter methylation exhibited significant association (*p = 0.04*) with the menopausal status of the female breast cancer patients, 65.38% (51/78) cases that showed promoter methylation were post-menopausal. Recent studies on Dysregulation of gene expression revealed the prominent role of epigenetics in gene silencing other than mutation, our study reveals *FOXO1* promoter methylation to be associated with low or no expression of *FOXO1* protein in breast cancer tissue in comparison to the adjacent normal tissue.

This study reports the tumor-suppressive role of FOXO1 in the case of Indian breast cancer patients and our data also suggested FOXO1 exhibited prognostic importance. Its downregulation is closely associated with the prognosis of the disease and different clinical parameters of the patients. We suggest that *FOXO1* can be taken as a biomarker in the case of breast cancer and further research can be carried out to find therapeutic strategies in targeting the *FOXO1* gene.

## Data Availability

The original contributions presented in the study are included in the article/Supplementary Material, further inquiries can be directed to the corresponding author.
